# Computationally Efficient 3D Orientation Tracking Using Gyroscope Measurements

**DOI:** 10.3390/s20082240

**Published:** 2020-04-15

**Authors:** Sara Stančin, Sašo Tomažič

**Affiliations:** Faculty of Electrical Engineering, University of Ljubljana, 1000 Ljubljana, Slovenia; saso.tomazic@fe.uni-lj.si

**Keywords:** computational efficiency, 3D gyroscope, 3D orientation, SORA, angular velocity, motion tracking

## Abstract

Computationally efficient 3D orientation (3DO) tracking using gyroscope angular velocity measurements enables a short execution time and low energy consumption for the computing device. These are essential requirements in today’s wearable device environments, which are characterized by limited resources and demands for high energy autonomy. We show that the computational efficiency of 3DO tracking is significantly improved by correctly interpreting each triplet of gyroscope measurements as simultaneous (using the rotation vector called the Simultaneous Orthogonal Rotation Angle, or SORA) rather than as sequential (using Euler angles) rotation. For an example rotation of 90°, depending on the change in the rotation axis, using Euler angles requires 35 to 78 times more measurement steps for comparable levels of accuracy, implying a higher sampling frequency and computational complexity. In general, the higher the demanded 3DO accuracy, the higher the computational advantage of using the SORA. Furthermore, we demonstrate that 12 to 14 times faster execution is achieved by adapting the SORA-based 3DO tracking to the architecture of the executing low-power ARM Cortex^®^ M0+ microcontroller using only integer arithmetic, lookup tables, and the small-angle approximation. Finally, we show that the computational efficiency is further improved by choosing the appropriate 3DO computational method. Using rotation matrices is 1.85 times faster than using rotation quaternions when 3DO calculations are performed for each measurement step. On the other hand, using rotation quaternions is 1.75 times faster when only the final 3DO result of several consecutive rotations is needed. We conclude that by adopting the presented practices, the clock frequency of a processor computing the 3DO can be significantly reduced. This substantially prolongs the energy autonomy of the device and enhances its usability in day-to-day measurement scenarios.

## 1. Introduction

By enabling 3D orientation (3DO) tracking, 3D gyroscopes have become an integral component of wearable devices used for ubiquitous motion capture, classification, and analysis [1,2,3,4,5,6,7,8,9,10,11,12,13,14,15,16,17,18,19,20,21]. In this context, resources are limited, and to prolong the energy autonomy of the measuring and processing device, efficient computations are required.

In this article, we investigate methods and present guidelines for achieving computationally efficient 3DO tracking. We consider a 3D gyroscope sensor that measures the angular velocities along its orthogonal sensitivity axes. In [22,23,24], we showed that these measurements are simultaneous and should be considered using the rotation vector called the Simultaneous Orthogonal Rotations Angle (SORA). In particular, in [22,23], we provided a mathematical derivation and numerical verification for SORA. In [24], we showed that, due to rotation non-commutativity, interpreting the measured rotations as sequential (i.e., Euler rotations) leads to a systematic error in the computed 3DO, and that this error grows with the angle of the actual rotation of the gyroscope. The smaller the rotation angle, the closer the rotations are to being commutative, and hence, the smaller the error in the computed 3DO. Therefore, it is not uncommon that for small angles, many still consider gyroscope measurements as sequential rotations. However, to obtain small rotation angles, high sampling frequencies are required, leading to a large number of measurements and consequently a large number of computational steps. In this article, we investigate this effect and compare the computational efficiency of both interpretations of gyroscope measurements at comparable levels of accuracy.

We also consider the computational efficiency of two commonly used methods for computing 3DO, i.e., the rotation matrix and rotation quaternions, when applied to gyroscope measurements. If the rotation matrix and quaternion are composed from the same rotation angle and axis, they are equivalent in terms of accuracy. Moreover, the rotation matrix can be transformed to the corresponding rotation quaternion and vice versa. However, these two methods differ with respect to computational efficiency. To provide a relevant comparison, we consider two application scenarios: when 3DO is calculated for each consecutive measurement step and when several consecutive measurements are combined into a single rotation composite, at which point the final 3DO is computed. The latter scenario is common and occurs when the sampling frequency, as dictated by the required accuracy, exceeds the rate at which the results are needed.

In addition to considering the effects of gyroscope measurement interpretations and 3DO computational methods, we investigate the possibilities of improving the computational efficiency by adapting the 3DO tracking implementation to the executing low-power microcontroller, which is suitable for achieving prolonged energy autonomy in the context of ubiquitous measurements and computations. In particular, we consider an implementation that uses only integer arithmetic and relies on lookup tables for evaluating the square and inverse square functions and small-angle approximations for evaluating trigonometric functions (as opposed to floating point arithmetic and library function calls).

The key contributions of this paper are the evaluations of computational efficiencies of different interpretations of gyroscope measurements, i.e., the simultaneous (using SORA) and sequential (using the Euler angles), different computational methods, i.e., rotation matrices and quaternions when used according to the simultaneous rotation interpretation, and different 3DO tracking implementations, i.e., implementation residing on integer arithmetic, lookup tables, and small-angle approximation as oppose to an implementation residing on floating point arithmetic and library function calls.

It is important to note that in this article, we assume that the sensor is suitably calibrated and that various sources of measurement inaccuracies, including output bias and inaccurate sensitivities and alignments of the sensor sensitivity axes are appropriately compensated. We also consider the observation interval to be sufficiently short for the sensor bias not to change significantly. The topic of sensor calibration and measurement error compensation has been extensively discussed in other literature reports, e.g., [25]. In addition, when the gyroscope sensors are combined with accelerometers in inertial measurement units (IMU), the Kalman filtering is a valuable technique that provides for data fusion and takes into account the correlations between measurements [26]. The Kalman filter is also commonly used for combining IMU and Global Positioning System (GPS) measurements [27] as well as with visual sensor measurements [28,29]. In this article, we focus our attention on gyroscope measurements exclusively. The methods presented are not an alternative to the Kalman filtering technique. However, the obtained conclusions can be incorporated in further advanced analysis that considers other sensors and is used to resolve other gyroscope-related problems than those presented here.

This article is organized as follows. We start our investigation by estimating the effects of the aforementioned computational efficiency influencers. In Section 2, we focus on the interpretation of gyroscope measurements; in Section 3, we focus on the 3DO computational methods; and in Section 4, we focus on the 3DO tracking implementation, which is adapted to the executing microcontroller. In Section 5, we present the experimental validation of the obtained theoretical results. Finally, in Section 6, we summarize our findings and draw conclusions.

In all the subsequent sections, we use the following notation rules: large bold letters denote matrices, small bold letters denote vectors, small bold italic letters denote quaternions, and large or small italics denote scalars.

## 2. Effect of 3D Gyroscope Measurement Interpretation on the Computational Efficiency

### 2.1. Noiseless Measurements

We consider a 3D gyroscope sensor that measures angular velocities around its three sensitivity axes *x*, *y*, and *z* at a sampling frequency *f_s_*. We further consider the 3DO to be computed after *M* consecutive measurements. The measurements are denoted as *ω_x,m_*, *ω_y,m_*, and *ω_z,m_* for the *x*, *y*, and *z* axes, respectively, and for each measurement step 1 ≤ *m* ≤ *M*, the angles of rotations around the sensor axes are given as follows:

(1) $$\varphi_{x,m}=\omega_{x,m}T_{s};\;\varphi_{y,m}=\omega_{y,m}T_{s};\;\varphi_{z,m}=\omega_{z,n}T_{s},$$

where *T_s_* denotes the sampling/measurement interval, i.e., *T_s_* = 1/*f_s_*.

In [22,23,24], we showed that the measured rotations are simultaneous and should be considered using the rotation vector SORA. As has been derived and illustrated in [22], the components of this vector are equal to the angles of three simultaneous rotations around orthogonal axes, while its magnitude and orientation are equal to the rotation angle and axis of a single equivalent rotation.

Following the mathematical derivation presented in [22], using the gyroscope measurements shown in Equation (1), the SORA vector for each measurement step *m*, denoted with $\Phi_{n}$ is given by:

(2) $$\Phi_{m}={\left[\begin{array}{ccc} \varphi_{x,m} & \varphi_{y,m} & \varphi_{z,m} \end{array}\right]}^{T}=T_{s}{\left[\begin{array}{ccc} \omega_{x,m} & \omega_{y,m} & \omega_{z,m} \end{array}\right]}^{T},$$

where ^T^ denotes the transpose operator. Furthermore, using *ϕ_m_* and **v***_m_* to denote the angle and axis of the equivalent rotation, respectively, for each *m*, we can write

(3) $$\Phi_{m}=\varphi_{m}v_{m}.$$

Thus, we can obtain *φ_m_* and **v***_m_* directly from gyroscope measurements as the magnitude and orientation of $\Phi_{m}$, respectively:

(4) $$\varphi_{m}=T_{s}\sqrt{\omega_{x,m}{}^{2}+\omega_{y,m}{}^{2}+\omega_{z,m}{}^{2}}\;=\sqrt{\varphi_{x,m}{}^{2}+\varphi_{y,m}{}^{2}+\varphi_{z,m}{}^{2}}$$

(5) $$v_{m}=\left[\begin{array}{c} v_{x,m}\\ v_{y,m}\\ v_{z,m} \end{array}\right]=\frac{1}{\sqrt{\varphi_{x,m}^{2}+\varphi_{y,m}^{2}+\varphi_{z,m}^{2}}}\left[\begin{array}{c} \varphi_{x,m}\\ \varphi_{y,m}\\ \varphi_{z,m} \end{array}\right]=\frac{1}{\sqrt{\omega_{x,m}^{2}+\omega_{y,m}^{2}+\omega_{z,m}^{2}}}\left[\begin{array}{c} \omega_{y,m}\\ \omega_{y,m}\\ \omega_{z,m} \end{array}\right].$$

It is important to note that for each measurement step *m*, the rotation axis **v***_m_* Equation (5) is given in the intrinsic coordinate system of the sensor.

As long as the axis of the actual rotation of the sensor does not change during the measurement interval *T_s_*, the equivalent rotation, given by Equations (2)–(5), is equal to the actual rotation of the sensor.

**Figure 1:**
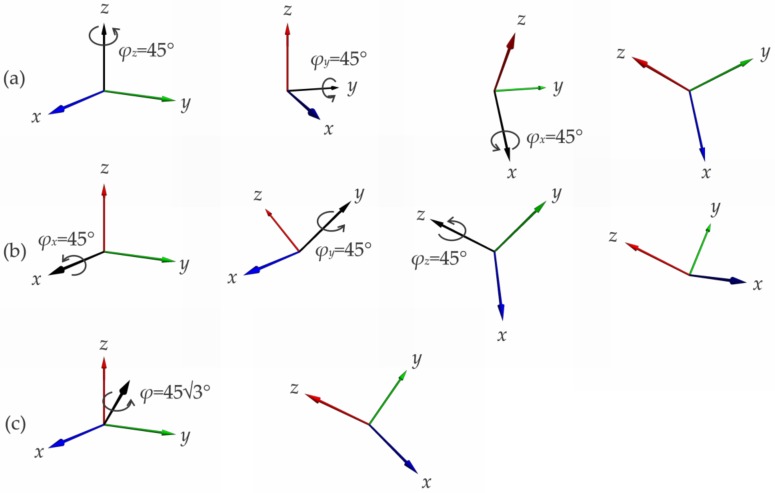
3D orientations (3DO) resulting from considering three simultaneous orthogonal rotations for 45° each as sequential rotations about in turn coordinate axes (**a**) *z*, *y*, and *x* and (**b**) *x*, *y*, and *z*. Due to the rotation non-commutativity, both result in a different 3DO and neither is equal to the actual 3DO of the sensor, which is illustrated with (**c**).

In contrast, as presented in [24], due to rotation non-commutativity, interpreting the three measured rotation angles in Equation (1) as sequential, i.e., Euler angles, leads to a systematic error in the estimated 3DO. Since the measurements in Equation (1) are given in the intrinsic coordinate system of the sensor, six different Euler sequences are possible: *x*-*y*-*z*, *y*-*z*-*x*, *z*-*x*-*y*, *x*-*z*-*y*, *z*-*y*-*x*, and *y*-*x*-*z*. In general, all six sequences result in a different 3DO, and in general, due to rotation non-commutativity, none of these results is equal to the actual 3DO of the sensor, even if the rotation axis does not change during the measurement interval *T_s_* [24]. An example of rotation non-noncommutativity and the error introduced with the sequential rotation interpretation is presented in Figure 1. In the presented example, a 3D gyroscope rotates about its intrinsic axis $\left[\begin{array}{ccc} 1 & 1 & 1 \end{array}\right]\prime /\sqrt{3}$ for an angle $45{\sqrt{3}}^{\circ }$ in a single measurement step. In such a scenario, the three angles measured are 45° each. As can be observed from Figure 1, considering the measured angles as sequential rotations in two different sequences, i.e., *z-y-x* and *x-y-z* brings to two different 3DO results. Neither of the obtained results is equal to the accurate 3DO of the rotated sensor, which is obtained by considering the three measured angles as simultaneous. For rotation angles of comparable magnitude, other Euler sequences introduce similar errors and give equally unreliable results.

As presented in [24], the systematic error introduced by the sequential rotation interpretation grows with the angle of the actual rotation of the gyroscope. To reduce this error, the rotation angle must be very small, implying a short measurement interval *T_s_*, i.e., a high sampling frequency *f_s_*. However, as we will demonstrate here, this approach limits the computational efficiency. In short, for a comparable accuracy of the computed 3DO, the sequential rotation interpretation requires a higher sampling frequency than does the simultaneous rotation interpretation, consequently leading to a larger number of necessary arithmetic operations for the same rotation time.

**Figure 2:**
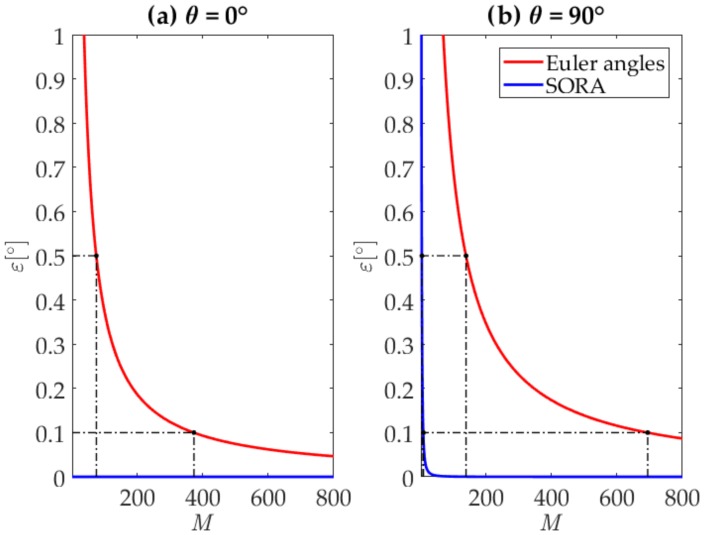
Required number of measurement steps for computing the 3D orientation (3DO) using the Simultaneous Orthogonal Rotation Angle (SORA) and Euler angles for noiseless measurements. The sensor rotates for an angle of *φ* = 90° around the axis **v** = [1 1 1]/√3. In example (**a**), the rotation axis is constant (*θ* = 0°), and using the SORA delivers accurate 3DO through a single measurement. Using Euler angles leads to a systematic error *ε* in the estimated 3DO, which decreases with the number of steps *M*. To achieve *ε* ≤ 0.5°, *M* ≥ 75 is required and for *ε* ≤ 0.1°, *M* ≥ 373 is needed. In example (**b**), the rotation axis itself rotates around the axis [1 0 0] for an angle of *θ* = 90°. Achieving *ε* ≤ 0.5° using the SORA requires *M* ≥ 4, and using Euler angles requires *M* ≥ 139 (34.75 times more than for the SORA). In the same example, for *ε* ≤ 0.1°, using the SORA requires *M* ≥ 9, and using Euler angles requires *M* ≥ 694 (77.11 times more than for the SORA).

The following example clearly demonstrates the computational advantage of using the SORA approach instead of the Euler angles method. Let us assume that the sensor rotates around the axis **v** = [1 1 1]/√3 for an angle of *φ* = 90°. Figure 2 shows the error in the estimated 3DO for both the SORA and Euler angles, which are calculated as presented in [24], as a function of the number of measurement steps *M*. In example (a), the rotation axis is constant, and in example (b), the rotation axis itself rotates uniformly during the time of observation around the axis [1 0 0] for an angle of *θ* = 90°.

During each measurement interval *m*, the sensor rotates for the angle *φ_m_ = φ*/*M*. Since the rotation axis itself rotates, we use the mean value (**v***_m_* + **v***_m_*_+1_)/2 for each *m* to obtain the SORA. For Euler angles, we consider rotations around in turn the *z*, *y*, and *x* axes, giving the aerospace sequence with rotation angles yaw, pitch, and roll equal to *ϕ_z,m_*, *ϕ_y,m_*, and *ϕ_x,m_*, respectively. We estimate the error *ε* as the smallest angle of a single rotation that would bring the sensor from the estimated to the true 3DO:

(6) $$\varepsilon =\mathrm{acos}((\mathrm{tr}(S^{T}S_{\mathrm{acc}})-1)/2),$$

where **S** denotes the 3 × 3 matrix of the estimated 3DO, using either SORA or Euler angles as presented in [24], and **S_acc_** denotes the 3 × 3 matrix of the accurate 3DO. The columns of these matrices are unit vectors representing the orientation of the intrinsic axes of the sensor in the reference coordinate system. Further on, ^T^ denotes the transpose operator and tr denotes the matrix trace operator, i.e., the sum of matrix diagonal elements.

We can observe that as long as the rotation axis is constant, using the SORA does not lead to any error in the computed 3DO and delivers accurate results through a single measurement. In contrast, using Euler angles leads to a systematic error in the computed 3DO; notably, for an ever-higher 3DO accuracy, an ever-higher number of measurement steps is required.

Introducing change in the rotation axis leads to an error in the computed 3DO for both the SORA and Euler angles; in general, the greater the change in the rotation axis, the greater the error and the more measurement steps are required to achieve the same accuracy. However, Euler angles require a significantly larger number of measurement steps. The higher the accuracy requirement, the greater the advantage of using the SORA. For the considered rotation example, for the 3DO error to not exceed the example margin of 0.5°, Euler angles require over 34 times more measurement steps than the SORA, and for the error to not exceed 0.1°, this ratio increases to 77.

### 2.2. Noisy Measurements

**Figure 3:**
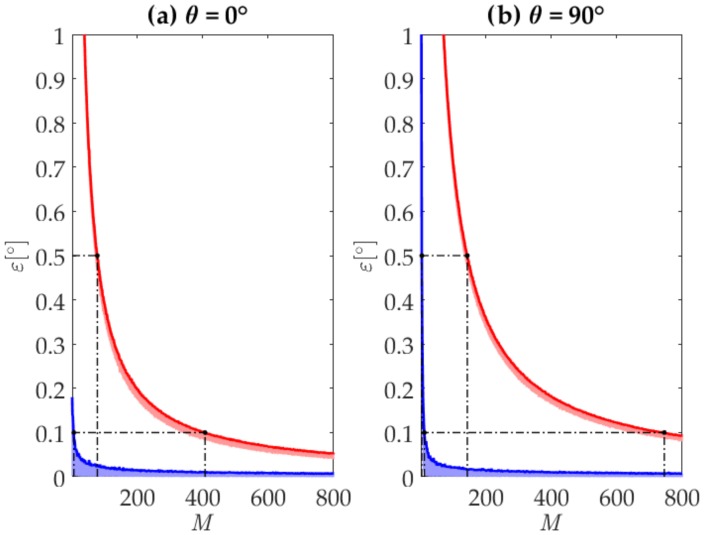
Required number of measurement steps for computing the 3D orientation (3DO) using the Simultaneous Orthogonal Rotation Angle (SORA) and Euler angles for noisy measurements. The sensor rotates for an angle of *φ* = 90° around the axis **v** = [1 1 1]/√3. One thousand repetitions of the experiment are considered for each number of measurement steps *M*. Due to noise, using both the SORA and Euler angles leads to a systematic error ε in the estimated 3DO, which decreases with the number of steps *M*. However, for comparable levels of accuracy, the SORA approach still significantly outperforms the Euler angles method. For a constant rotation axis (example (**a**) *θ* = 0°) and using the SORA, *ε* ≤ 0.5° is achieved through a single measurement, and *ε* ≤ 0.1° is obtained in *M* ≥ 6 steps. Using Euler angles requires *M* ≥ 78 for *ε* ≤ 0.5° and *M* ≥ 407 for *ε* ≤ 0.1°. For example, (**b**), the rotation axis itself rotates around the axis [1 0 0] for an angle of *θ* = 90°. Achieving *ε* ≤ 0.5° using the SORA requires *M* ≥ 4, and using Euler angles requires *M* ≥ 143 (35.75 times more than for the SORA). In the same example, for *ε* ≤ 0.1°, using the SORA requires *M* ≥ 12, and using Euler angles requires *M* ≥ 745 (62.08 times more than for the SORA).

In the presence of measurement noise at reasonable levels, the computational advantage of the SORA versus Euler angles does not change, as illustrated in Figure 3. Zero-mean Gaussian noise with a standard deviation of 0.05°/s was added to the angular velocities, which conforms to the experimentally determined distribution of noise for the 3D gyroscope in the MPU-6500 device manufactured by InvenSense, San Jose, California [30]. To evaluate the impact of noise on the accuracy, we performed 1000 repetitions of the experiment for each number of measurement steps *M*.

The results show that using Euler angles requires 35 times more measurement steps to achieve *ε* ≤ 0.5° and 78 times more measurement steps to achieve *ε* ≤ 0.1°.

The presented example clearly illustrates that with respect to computational efficiency, the SORA approach outperforms the Euler angle method. Thus, only the use of the SORA is considered for computationally efficient 3DO tracking in the remainder of this paper.

## 3. Effect of the Computational Method on the Computational Efficiency

Two computational methods are common for tracking 3DO using gyroscope measurements: the rotation matrix and quaternion. If composed using the same rotation axis and angle, both the rotation matrix and quaternion are equivalent in terms of accuracy but differ in the number of arithmetic operations they require.

In the remaining section, we present only the equations necessary for tracking 3DO. Both methods are commonly known in the field, and more detailed presentations than presented here are available from numerous sources, such as [31,32].

To express the 3DO, we introduce the 3 × 3 matrices **S_0_** and **S**. The columns of these matrices are unit vectors representing the orientation of the intrinsic axes of the sensor in the reference coordinate system, and the rows are unit vectors representing the orientation of the reference axes in the coordinate system of the sensor. **S_0_** represents the initial orientation, and **S** represents the resulting orientation, which is computed by treating the gyroscope measurements as simultaneous rotations.

### 3.1. DO Computation

#### 3.1.1. Rotation Matrix

For each step measurement *n*, the rotation matrix **R***_m_* can be expressed using the rotation angle *φ_m_* Equation (4) and axis **v***_m_* Equation (5) as

(7) $$R_{m}=\left[\begin{array}{ccc} c_{m}+v_{x,m}^{2}(1-c_{m}) & v_{x,m}v_{y,m}(1-c_{m})-v_{z,m}s_{m} & v_{x,m}v_{z,m}(1-c_{m})+v_{y,m}s_{m}\\ v_{x,m}v_{y,m}(1-c_{m})+v_{z,m}s_{m} & c_{m}+v_{y,m}^{2}(1-c_{m}) & v_{y,m}v_{z,m}(1-c_{m})-v_{x,m}s_{m}\\ v_{x,m}v_{z,m}(1-c_{m})-v_{y,m}s_{m} & v_{y,m}v_{z,m}(1-c_{m})+v_{x,m}s_{m} & c_{m}+v_{z,m}^{2}(1-c_{m}) \end{array}\right],$$

where *c_m_* and *s_m_* denote cos(*φ_m_*) and sin(*φ_m_*), respectively.

After *M* consecutive rotations, 3DO is given by

(8) $$S=S_{0}R_{M}\cdots R_{m}\cdots R_{1}=S_{0}R,$$

where **R** denotes the composite rotation matrix, which combines the effects of all *M* measured rotations.

The order of matrix multiplication in Equation (8) accounts for the fact that the axis of rotation **v***_m_* Equation (5) is given in the intrinsic coordinate system of the sensor, which corresponds to the observation of the reference system rotating in the coordinate system of the sensor for the angle –*φ_m_* Equation (4).

#### 3.1.2. Rotation Quaternion

For each measurement step *m*, the rotation quaternion ***q****_m_* with elements *q_m,_*_0_, *q_m,_*_i_, *q_m,_*_j_, and *q_m,_*_k_ can be expressed using the rotation angle *φ_m_* Equation (4) and axis **v***_m_* Equation (5) as follows:

(9) $$q_{m}=q_{m,0}+q_{m,i}i+q_{m,j}j+q_{m,k}k=\cos(\varphi_{m}/2)+\sin(\varphi_{m}/2)\left(v_{x,m}i+v_{y,m}j+v_{z,m}k\right),$$

where **i**, **j**, and **k** are fundamental quaternion units, giving the standard orthonormal basis for R^3^ and satisfying the following fundamental rules:

(10) $$i^{2}=j^{2}=k^{2}=ijk=-1,\;ij=k=-ji;\;jk=i=-kj;\;ki=j=-ik.$$

The final 3DO can be obtained by rotating each of the three reference axes in the intrinsic coordinate system of the sensor for the angle –*φ_m_* Equation (4) around the axis **v***_m_* Equation (5) for each measurement step *m*. Let us use ***s_0_****_r_* and ***s****_r_* to denote vector quaternions, i.e., quaternions with the first, scalar component equal to 0, and with vector elements equal to the elements of the *r*th rows of matrices **S_0_** and **S**, respectively:

(11) $$s_{0r}=0+S_{0}{}_{r,1}i+S_{0}{}_{r,2}j+S_{0}{}_{r,3}k,\;s_{r}=0+S_{r,1}i+S_{r,2}j+S_{r,3}k;\;1\leq r\leq 3.$$

Quaternions ***s_0_****_r_* and ***s****_r_* represent the orientation of the reference axes in the coordinate system of the sensor before and after rotation, respectively. We can write

(12) $$\begin{array}{ll} s_{r} & =q_{M}^{*}\cdots q_{m}^{*}\cdots q_{1}^{*}\;s_{0r}\;q_{1}\cdots q_{m}^{}\cdots q_{M}^{}\\ & =(q_{1}\cdots q_{m}^{}\cdots q_{M}^{}){\;}^{*}s_{0r}\;q_{1}\cdots q_{m}^{}\cdots q_{M}^{}\\ & =q_{}^{*}s_{0r}\;q. \end{array}$$

In Equation (12), ***q*** denotes the composite rotation quaternion combining the effects of all *M* measured rotations, $q_{}^{*}$ is its composite, and $q_{m}^{*}$ is the complex conjugate of the *m*th rotation quaternion $q_{m}$.

The quaternion multiplication in Equation (12) is performed according to the Hamiltonian product, i.e., in a component-wise manner. The order of quaternion multiplication in Equation (12) accounts for the fact that the axis of rotation **v***_m_* in Equation (5) is given in the intrinsic coordinate system of the sensor.

By computing all three rows of **S** according to Equation (12), the 3DO of the sensor is fully known.

At this point, let us emphasize that the composite rotation quaternion ***q*** can be transformed into the rotation matrix **R**. As will be demonstrated in the next subsection, transforming ***q*** to **R** and then using Equation (8) to compute 3DO is computationally more efficient than using Equation (12) directly. Denoting the elements of ***q*** as *q*_0_, *q*_i_, *q*_j_, and *q*_k_, the following relation holds:

(13) $$R=\left[\begin{array}{ccc} 1-2q_{j}^{2}-2q_{k}^{2} & 2q_{i}^{}q_{j}-2q_{0}q_{k} & 2q_{0}q_{j}+2q_{i}q_{k}\\ 2q_{i}q_{j}+2q_{0}q_{k} & 1-2q_{i}^{2}-2q_{k}^{2} & -2q_{0}q_{i}+2q_{j}q_{k}\\ -2q_{0}q_{j}+2q_{i}q_{k} & 2q_{0}q_{i}+2q_{j}q_{k} & 1-2q_{i}^{2}-2q_{j}^{2} \end{array}\right].$$

### 3.2. Computational Efficiency

To estimate the computational efficiency, we divide the operations required to compute the 3DO, using either of the two methods, into the following four categories: additions and subtractions (**A**), general multiplications (**M**), vector normalization (**VN**), and mathematical functions, including the square root and trigonometric value estimations (**F**).

Obviously, to achieve computational efficiency, the implementations of all of the above-mentioned operations should be adapted to the architecture of the executing device. Hence, due to the high computational complexity of the division operation, we implement vector normalization as one inverse function evaluation and three multiplications. Furthermore, we implement multiplications by 2 and 2^−1^ as left and right bit shift operations, respectively, as these operations are computationally less expensive than are general multiplications. It is also important to note that for estimating the computational efficiency, we neglect multiplications by 2 and 2^−1^ altogether, since their numbers are negligible in comparison to the number of general multiplications when computing the 3DO.

#### 3.2.1. Common Operations

Computing the rotation angle *φ_m_* and axis **v***_m_* from the measured angular velocities according to Equations (4) and (5), respectively, requires two (**A**), four (**M**), one (**VN**), and one (**F**) for each measurement step *m*, giving 2 *M* (**A**), 4 *M* (**M**), 1 *M* (**VN**), and 1 *M* (**F**) for *M* measurement steps. These operations are added to the operations specific to the rotation matrix and the rotation quaternion computational methods.

#### 3.2.2. Rotation Matrix

Assuming that the results of the repeating products are stored in memory, composing each rotation matrix **R***_m_* Equation (7) requires 10 (**A**), 12 (**M**), and 2 (**F**) operations, giving, together with the previously established number of operations needed to compute the rotation angle and axis, 12 *M* (**A**), 16 *M* (**M**), 1 *M* (**VN**), and 3 *M* (**F**) operations for all *M* rotation matrices. According to Equation (8), the 3DO is calculated by multiplying *M* 3 × 3 matrices, thus further requiring 18 *M* (**A**) and 27 *M* (**M**) operations.

Adding all of the operations together gives 30 *M* (**A**), 43 *M* (**M**), 1 *M* (**VN**), and 3 *M* (**F**) operations required for *M* measurement steps.

#### 3.2.3. Rotation Quaternion

Composing each rotation quaternion $q_{m}^{}$ according to Equation (9) requires 3 (**M**) and 2 (**F**), giving, together with the established number of operations needed to calculate the rotation axis and angle, 2 *M* (**A**), 7 *M* (**M**), 1 *M* (**VN**), and 3 *M* (**F**) operations for all *M* rotation quaternions.

The quaternion multiplication process conforms to the component-wise rule and requires 12 (**A**) and 16 (**M**) operations. For the *M* consecutive rotations in Equation (12), *M*-1 such multiplications are needed, resulting in 12 (*M*-1) (**A**) and 16 (*M*-1) (**M**) operations. From this result, we can conclude that computing ***q*** requires 14 *M*-12 (**A**), 23 *M*-16 (**M**), 1 *M* (**VN**), and 3 *M* (**F**). After ***q*** is computed, its complex conjugate is also known and does not require any additional operations.

Using the composite rotation quaternion to rotate a single axis according to Equation (12) requires two quaternion multiplications. Taking into account that the first multiplication, i.e., $q_{}^{*}s_{0r}$ is between a general quaternion and a vector quaternion and that the final result is a vector quaternion, we can determine that a total of 17 (**A**) and 24 (**M**) operations are required for each axis. When rotating all three axes, the number of previously established operations triples, giving 51 (**A**) and 72 (**M**) operations. Adding all of the operations together gives 14 *M* + 39 (**A**), 23 *M* + 56, 1 *M* (**VN**), and 3 *M* (**F**) operations required for *M* measurement steps.

### 3.3. Discussion

The established computational efficiencies for both computational methods when used according to the simultaneous rotation interpretation (SORA) are concisely presented and provided for comparison in Table 1. Having an equal number of (**VN**) and (**F**), the rotation matrix and the rotation quaternion computational methods differ only with respect to the number of (**A**) and (**M**) operations.

For the simultaneous rotation interpretation, we can conclude that when the 3DO is computed for each consecutive measurement step (*M* = 1), the rotation matrix method computationally outperforms the rotation quaternion approach, requiring 23 (**A**) and 36 (**M**) fewer operations per each step *m*. In a relative measure, this advantage is equal to 1.77 and 1.84 times fewer number of (**A**) and (**M**) operations, respectively.

When, on the other hand, several consecutive measurements are combined into a single rotation composite, at which point only the final 3DO is computed (*M* >> 1), the rotation quaternions computationally significantly outperform the matrices, requiring 16 *M*-39 (**A**) and 20 *M*-56 (**M**) fewer operations. In a relative measure, for large values of *M*, this advantage is equal to 2.14 and 1.87 times fewer numbers of (**A**) and (**M**) operations, respectively.

A 3DO tracking scenario relying only on the final 3DO result of a composite of *M* rotations is common and typically occurs when the measurement sampling frequency, as dictated by the required 3DO accuracy [24], exceeds the rate at which the 3DO results are needed and used. When this is the case, all *M* rotations can be conveniently combined and represented with either a single rotation matrix or a quaternion composite. Both forms accurately represent the cumulative effect of all *M* rotations. However, although rotation matrices provide a computationally more efficient 3DO computation, rotation quaternions provide a more efficient rotation composite computation; specifically, multiplying two rotation matrices requires 18 (**A**) and 27 (**M**) operations, and multiplying two rotation quaternions requires only 12 (**A**) and 16 (**M**) operations. For this reason, the effects of all *M* consecutive measurement steps should be combined into a composite rotation quaternion.

Finally, as a minor addition to optimization, the composite rotation quaternion should be transformed into the rotation matrix before the final 3DO computation. Namely, as we have already noted, it is computationally more efficient to transform ***q*** to **R** and then use Equation (8) to compute the 3DO than to use Equation (12) directly. Assuming that the results of the repeating products in Equation (13) are stored in memory and that multiplications by 2 are neglected, transforming ***q*** to **R** requires 12 (**A**) and 9 (**M**) operations. Using then **R** to compute the 3DO according to Equation (8), as has been previously established, requires an additional 18 (**A**) and 27 (**M**) operations; together, this gives only 30 (**A**) and 36 (**M**) operations as opposed to 51 (**A**) and 72 (**M**) needed when ***q*** is used directly.

Adding the initially established operations required to compute ***q***, to transform it to the rotation matrix **R**, and finally to use **R** to obtain the 3DO gives a total of 14 *M* + 18 (**A**), 23 *M* + 20 (**M**), 1 *M* (**VN**), and 3 *M* (**F**) operations required for *M* measurement steps. Requiring 16 *M*-18 (**A**) and 20 *M*-20 (**M**) fewer operations, this approach outperforms the rotation matrix method for all *M* > 1.

When using rotation quaternions for all composites consisting of *M* > 1 rotations, it is hence reasonable to transform the quaternion composite into the rotation matrix, and we do so in all the examinations in the remainder of this paper.

For comparison, computational efficiencies of both computational methods when used according to the sequential rotations interpretation (Euler angles), established in a similar way and by following equations presented in [24] are also presented in Table 1. Comparing the results for both interpretations, we can conclude that for rotation matrices, the Euler angles outperform SORA with respect to (**A**), (**M**), and (**VN**) operations, while SORA requires fewer (**F**) operations. On the other hand, for rotation quaternions, SORA outperforms the Euler angles with respect to (**A**), (**M**), and (**F**) operations, while Euler angles do not require 1 *M* (**VN**) operations.

However, this comparison is valid only in the case of equal number of measurement steps *M* for both methods. As has been established in Section 2, for results of comparable accuracy, in general, Euler angles require a significantly larger number of measurement steps. The computational complexity associated with this larger number of measurement steps is significantly higher than the differences between SORA and the Euler angles presented in Table 1. Thus, we can conclude that, for a given accuracy level, the difference between computational complexities between SORA and Euler angles follows the results presented in Section 2. On the other hand, an intra-interpretation comparison of computational complexities, as both computational methods for a particular interpretation give equal 3DO results, follows the results presented in Table 1.

## 4. Effect of 3DO Tracking Implementation on the Computational Efficiency

In addition to the number of operations required to compute the 3DO, as presented in the previous section, the actual 3DO computation time depends strongly on the architecture of the computing device. To ensure sufficient energy autonomy in the context of ubiquitous measurements, we investigate the possibilities of improving the computational efficiency by adapting the 3DO tracking implementation to a computing low-power microcontroller. In particular, we considered the Arduino Genuino Zero board [33], which contains a 32-bit ARM Cortex^®^ M0+ microcontroller developed by Atmel [34]. The microcontroller features a 48 MHz clock, 32 KB static random-access memory (SRAM), and 256 KB flash memory. The power consumption of the integrated microcontroller is sufficiently low enough to be appropriate in a context where energy autonomy is essential, such as in ubiquitous measurements and computing using wearable devices.

Since the board we used for the experiment does not include a floating-point arithmetic unit, we used integer arithmetic exclusively. We scaled all the intermediate results by as large a power of two as possible to take full advantage of the 32-bit integer data type associated with the used 32-bit microcontroller and by considering the limited range and specified accuracy of the numerical values at a specific level of 3DO computation. In this manner, we avoided general and expensive floating-point-to-integer data-type conversions. With such an implementation, we achieved greater computational efficiency for each of the tested 3DO computational methods, which consequently led to more reliable and meaningful validation.

To increase the computational efficiency, we implemented the vector normalizations in Equation (5) as multiplications with the inverse value of the vector norm, i.e., square root value. The square root function used for computing the angle of rotation in Equation (4) and the inverse square function used for vector normalization were both implemented as a combination of a lookup table and linear interpolation. Both lookup tables, the square root and the inverse square root, were designed to give the highest accuracy and still fit into the available SRAM of the microcontroller.

We considered the measurement accuracy to be limited to two decimal places. We also considered a limited range of the measured angular velocity values in the range of $0\leq \omega_{m}=\sqrt{\omega_{x,m}^{2}+\omega_{y,m}^{2}+\omega_{z,m}^{2}}<75.00^{\circ }\;s^{-1}.$ The lookup tables contained the square root and inverse square root values for 3434 equidistant points from the interval [0 (75.00° s^−1^)^2^>. The values between these points were estimated using linear interpolation. The mean errors obtained for the square root and inverse square root implementations for the specified limited range of the input angular velocity values were 0.003° s^−1^ and 0.0001° s^−1^, respectively. Both mean errors were so below the considered accuracy of the input angular velocity, which was set to two decimal places.

Both lookup tables were stored in SRAM. Containing values for 3434 equidistant points, each occupied 3434·4 B = 13.736 KB of SRAM. Implementing both lookup tables left 32 KB − 2·13.736 KB = 4.528 KB free for the other requirements of the 3DO computation. The limited range of input angular velocity values was sufficient for the testing purpose presented in this article. For higher angular velocity values in a particular measurement scenario, for the same level of 3DO accuracy, we would use more KB of working memory and implement larger lookup tables. An alternative approach would be to reduce the result accuracy. Both changing the angular velocity range and result accuracy is easily made by modifying the corresponding variables in the code and can be achieved prior to every measurement.

Furthermore, for the sampling frequencies that are common for devices as was the one used here, the measured rotation angles are small. For example, for a sampling frequency *f_s_* = 100 Hz, the rotation angle for the considered limited input angular velocity range does not exceed 75.00° s^−1^ · 0.1 s = 0.75°. Considering this, we implemented the sine and cosine function evaluations using the small-angle approximation.

Following the aforementioned guidelines, we implemented the 3DO computation in the C/C++ Arduino IDE environment in accordance with the equations presented in Section 3. An experimental validation showed that the execution time for this implementation is significantly shorter than that for an implementation based on floating-point arithmetic and library function calls (for estimating the square, inverse square, sine and cosine function values): 14 times shorter for the rotation matrix method and 12 times shorter for the rotation quaternion approach.

The increase in efficiency was partially achieved at the expense of numerical accuracy. However, multiple tests performed showed that the obtained 3DO results after one rotation were accurate up to the fifth decimal for the entire range of input angular velocity values. The 3DO results after 150 rotations were accurate up to the second decimal place. This level of accuracy is well within the acceptable range and satisfies the reasonable objectives of our investigation. The accuracy was validated using a 64-bit Intel i7-700 processor, floating-point arithmetic, and library function calls.

The original code developed is provided in the Supplementary Files as well as in a GitHub repository [35].

## 5. Experimental Validation

### 5.1. Experiment

To evaluate the implications of the established computational efficiencies for the execution time, we designed the following 3DO tracking experiment. We considered simulated random angular velocity measurements for 10,000 consecutive measurement steps. We considered two different 3DO computation scenarios:

- stepwise 3DO computation, in which we computed the 3DO for each consecutive measurement step, setting so *M* = 1 and performing 10,000 3DO computations, and
- only the final 3DO computation, in which we combined several consecutive measurements into a single rotation composite, at which point we computed the final 3DO setting so that *M* = 10,000 and performing a single 3DO computation.

We performed all 3DO computations using the SORA. For both scenarios, we considered both computational methods, the rotation matrix and quaternion, considering the equations presented in Section 3 and implemented and executed using the Arduino Genuino Zero board, as presented in Section 4.

We measured the execution times using the integrated Arduino library function micros() calls. The micros() function returns the time in microseconds, since the Arduino board began running the current program.

### 5.2. Results and Discussion

The results are presented in Table 2; they confirm that for (a) stepwise computation, the rotation matrix method outperforms the rotation quaternion approach. Using the rotation quaternions, the 3DO was computed in 561.4 ms, and using the rotation matrices required 304.2 ms, reducing the execution time for a factor of 1.85.

The execution time for rotation matrices for (b) was equal to the execution time for (a), reflecting the same number of operations that are required when using rotation matrices in both scenarios. However, using rotation quaternions for (b) significantly reduced the execution time, i.e., to 173.7 ms, outperforming the rotation matrix approach for a factor of 1.75.

Multiple repetitions of the experiment for different numbers of consecutive measurements and input angular velocity values yielded similar results.

We can further conclude that both computational methods support stepwise 3DO computations in real time using the 32-bit ARM Cortex^®^ M0+ microcontroller. In both scenarios, both methods required less than 0.1 ms for the computation per each measurement step, which is negligible compared to the sampling interval of 10 ms at a sampling frequency of 100 Hz. However, the high computational efficiency of the 3DO estimation allows the processor to simultaneously compute the 3DO values of multiple gyroscopes and perform other tasks. In addition, high computational efficiency enables the use of processors with a slow clock, which significantly reduces the energy consumption and thus prolongs the energy autonomy of the device.

## 6. Conclusions

We presented practices for improving the computational efficiency of tracking 3DO using gyroscope angular velocity measurements.

First, by correctly interpreting the measurements as simultaneous (using the SORA) rather than as sequential rotations (using Euler angles), we showed that the 3DO results are more accurate, and the computation is more efficient. For the simple illustrative example considered in which the sensor rotates for an angle of *φ* = 90°, Euler angles have been shown to require significantly more measurement steps for comparable levels of result accuracy. For the 3DO error not to exceed 0.5°, 78 times more measurement steps were required for a fixed rotation axis and 35 times more measurement steps were required for a rotation axis, which itself uniformly rotates during the time of observation.

Second, we demonstrated that the computational efficiency could be further increased by adapting the SORA-based 3DO tracking to the architecture of the computing device. On a 32-bit ARM Cortex^®^ M0+ microcontroller, the implementation scheme, which relies on integer arithmetic, lookup tables for the square root and inverse square root functions, and small-angle approximations for cosine and sine functions, performs computations 12 to 14 times faster than an implementation that uses floating-point arithmetic and library function calls.

Finally, we showed that the execution time could be additionally reduced by applying the appropriate computational method using optimized SORA-based 3DO tracking. By measuring and comparing the execution times using simulated measurements, we concluded that both computational methods, rotation matrices and quaternions, are well suited for real-time 3DO tracking with the specific 32-bit ARM Cortex^®^ M0+ microcontroller. However, the obtained experimental results showed that the rotation matrix method performs 1.85 times faster when the 3DO is estimated for each consecutive measurement step. On the other hand, the rotation quaternion approach performs 1.75 times faster when several consecutive measurements are combined into a single rotation composite, at which point only the final 3DO is required.

Experimental results confirmed that by choosing an appropriate interpretation of gyroscope measurements and an appropriate computational method with an efficient implementation scheme, we could significantly reduce the computational time.

We can finally conclude that by adopting the practices and guidelines presented in this paper, the clock frequency of a processor computing the 3DO can be significantly reduced. Substantially prolonging energy autonomy and enhancing usability in day-to-day measurement scenarios, this is especially important in the context of wearable devices.

The conclusions obtained can be incorporated into an extended analysis, which also considers other sensors and is used to solve gyroscope-related problems other than the ones presented here, and they may be the subject of further research.

## Figures and Tables

**Table 1:** Computational efficiency of rotation matrices and quaternions for computing the 3D orientation (3DO) after *M* consecutive measurement steps.

Interpretation	Computational Method	Computational Efficiency ^1^			
		(A)	(M)	(VN)	(F)
simultaneous ^2^	rotation matrix	30 *M*	43 *M*	1 *M*	3 *M*
	rotation quaternion	14 *M* + 39	23 *M* + 56	1 *M*	3 *M*
sequential ^3^	rotation matrix	22 *M*	42 *M*	0	6 *M*
	rotation quaternion	16 *M* + 39	31 *M* + 56	0	6 *M*

^1^ Measured as the number of operations: (**A**)—additions and subtractions (**M**)—general multiplications (**VN**)—3D vector normalizations (**F**)—mathematical functions, including the square root and trigonometric value estimations. ^2^ Three orthogonal rotations measured with a 3D gyroscope interpreted as simultaneous rotations (using SORA. ^3^ Three orthogonal rotations measured with a 3D gyroscope interpreted as sequential, i.e., Euler rotations.

**Table 2:** Execution times of tracking 3D orientation (3DO) for 10,000 consecutive rotation measurements.

Computational Scenario	Computational Method	Execution Time *T* [ms]	Gain Factor ^3^
Stepwise ^1^	Rotation matrix	304.2	1.85
	Rotation quaternion	561.4	1.00
Only final ^2^	Rotation matrix	304.2	1.00
	Rotation quaternion	173.7	1.75

^1^ 3DO is computed for each consecutive measurement step. ^2^ All consecutive measurements are combined into a single composite, at which point only the final 3DO is computed. ^3^ The gain factor is computed as the ratio between the longest execution time obtained (rotation quaternion approach for stepwise and rotation matrix method for only the final 3DO computation) and the execution time for the specific computational method.

## Supplementary Materials

The following are available online at https://www.mdpi.com/1424-8220/20/8/2240/s1, Figure S1: 3D orientation error resulting from interpreting the three gyroscope measurements as sequential rotations, Figure S2: Required number of measurement steps for computing the 3D orientation using the Simultaneous Orthogonal Rotation Angle (SORA) and Euler angles for noiseless measurements, Figure S3: Required number of measurement steps for computing the 3D orientation using the Simultaneous Orthogonal Rotation Angle (SORA) and Euler angles for noisy measurements, Table S1: Computational efficiency of rotation matrices and quaternions for computing the 3D orientation (3DO) after M consecutive measurement steps, Table S2: Execution times of tracking 3D orientation (3DO) for 10,000 consecutive rotation measurements, Source code S1: Computationally efficient 3D orientation tracking.
